# Development of Sustained Release “NanoFDC (Fixed Dose Combination)” for Hypertension – An Experimental Study

**DOI:** 10.1371/journal.pone.0128208

**Published:** 2015-06-05

**Authors:** Anjuman Arora, Nusrat Shafiq, Sanjay Jain, G. K. Khuller, Sadhana Sharma, Samir Malhotra

**Affiliations:** 1 Dept. of Pharmacology, Post Graduate Institute of Medical Education and Research, Chandigarh, India; 2 Dept. of Internal Medicine, Post Graduate Institute of Medical Education and Research, Chandigarh, India; 3 Dept. of Biochemistry, Post Graduate Institute of Medical Education and Research, Chandigarh, India; Tohoku University, JAPAN

## Abstract

**Objectives:**

The present study was planned to formulate, characterize and evaluate the pharmacokinetics of a novel “NanoFDC” comprising three commonly prescribed anti-hypertensive drugs, hydrochlorothiazide (a diuretic), candesartan (ARB) and amlodipine (a calcium channel blocker).

**Basic Methods:**

The candidate drugs were loaded in Poly (DL-lactide-co-gycolide) (PLGA) by emulsion- diffusion-evaporation method. The formulations were evaluated for their size, morphology, drug loading and *in vitro* release individually. Single dose pharmacokinetic profiles of the nanoformulations alone and in combination, as a NanoFDC, were evaluated in Wistar rats.

**Results:**

The candidate drugs encapsulated inside PLGA showed entrapment efficiencies ranging from 30%, 33.5% and 32% for hydrochlorothiazide, candesartan and amlodipine respectively. The nanoparticles ranged in size from 110 to 180 nm. *In vitro* release profile of the nanoformulation showed 100% release by day 6 in the physiological pH 7.4 set up with PBS (phosphate buffer saline) and by day 4-5 in the intestinal pH 1.2 and 8.0 set up SGF (simulated gastric fluid) and SIF (simulated intestinal fluid) respectively. In pharmacokinetic analysis a sustained-release for 6 days and significant increase in the mean residence time (MRT), as compared to the respective free drugs was noted [MRT of amlodipine, hydrochlorothiazide and candesartan changed from 8.9 to 80.59 hours, 11 to 69.20 hours and 9 to 101.49 hours respectively].

**Conclusion:**

We have shown for the first time that encapsulating amlodipine, hydrochlorothiazide and candesartan into a single nanoformulation, to get the “NanoFDC (Fixed Dose Combination)” is a feasible strategy which aims to decrease pill burden.

## Introduction

Hypertension is one of the most common conditions in primary care and one of the key risk factors, along with hyperlipidemia, hyperglycemia, obesity and smoking, etc., that contribute to other diseases like myocardial infarction, stroke, renal failure and death.[1] Joint National Committee VIII states that there are more than 1 billion hypertensive patients world-wide.[2] As per the WHO report on World Health Statistics 2012, one in every three adults has raised blood pressure.[3]

Randomized clinical trials have time and again shown the benefit of anti-hypertensive drug treatment in reducing blood pressure. According to the current guidelines, any one of the drug classes among Diuretics like Hydrochlorothiazide or Aldosterone blockers like spironolactone, Angiotensin-converting enzyme (ACE) inhibitors like enalapril or Angiotensin receptor blockers (ARB) like candesartan, Beta blockers like atenolol, Calcium channel blockers like nifedipine, can be started. However, it is well known that a large no. of patients fail to respond to a single drug. A significant number of patients, particularly those at high risk, need three or more agents to achieve blood pressure goals.[4] International guidelines also recommend combination therapy in clinical practice of arterial hypertension, even as a first-line strategy in patients with high or very high cardiovascular risk.[5,6,7,8] Certain combination therapies, like ACE inhibitors and thiazide diuretics, Angiotensin II receptor blockers and thiazide diuretics, ACE inhibitors and dihydropyridine calcium antagonists, Angiotensin II receptor blockers and dihydropyridine calcium antagonists, have a considerable evidence in the treatment of hypertension.[7]

Due to the chronic nature of the treatment and possibility of side-effects of the drugs being taken, problems like decreased adherence to the treatment and thus suboptimal blood pressure control, organ damage and cardiovascular complications surface.[9,10]

Various approaches have been proposed and tried, to improve compliance to hypertension therapy. The use of a fixed-dose combination therapy, targeting several risk factors, in the form of a ‘polypill’, was first proposed by Wald and Law as a concept in 2003.[11,12] The fixed-dose combination is foreseen to lead to improvement in the patient compliance, reduced cost and ease of use by the elderly. Study groups across the world have shown these benefits from FDCs.[13, 14, 15, 16] Other than this, there are many approaches to deliver combination of drugs via the multi-drug delivery systems. There are many already available for cancer therapy.[17] There advantages in efficacy and lowering of side effects have been well demonstrated. [18]

An extension of the concept of polypill would be a sustained release polypill. Previous work by our group on first line (rifampicin, isoniazid, pyrazinamide and ethambutol) [19] and second-line (ethionamide and levofloxacin) [20,21] antitubercular drugs encapsulated inside PLGA nanoparticles leads to a sustained release of the drug in plasma for up to 7 days. PGA, PLA and their co-polymer PLGA, are the most commonly used family of biodegradable and biocompatible polymers because of its several advantages. Some of these include its ability to entrap a wide range of hydrophobic or hydrophilic drugs inside nano sized particles by choosing the different ratios of the glycolic and lactic acid monomers. It is also approved by the US-FDA for human use.[22] The PLGA based nanoparticles have shown their advantage over liposomes by their increased stability and the unique ability to create an extended release. [23]

With this background we planned to develop, characterize, optimize and evaluate a “NanoFDC” with a combination of three commonly co-prescribed anti-hypertensive drugs, namely hydrochlorothiazide, amlodipine and candesartan, encapsulated inside PLGA nanoparticles. The novelty of this “NanoFDC” will be that it includes the three most commonly co-prescribed anti-hypertensive drugs, together, encapsulated inside PLGA nanoparticles separately, so as to achieve a sustained release for over a week. Such a combination is not being worked on. We have tested and proved this concept as feasible in our IHD “Nanopolypill” for the first time. [24]

## Methods

### Materials

Poly (DL-lactide-co-gycolide) (PLGA) (50:50 resomer) was purchased from Birmingham Polymers, Inc (Birmingham, AL). Amlodipine was obtained from IPCA Laboratories Ltd. (Mumbai, India), hydrochlorothiazide from Unichem laboratories Pvt. Ltd. Mumbai, India, and candesartan was received as a gift from Stancare, Ranbaxy Laboratories Ltd. (Gurgaon, India). Poly Vinyl Alcohol (PVA, MW 30000–70000 Da, 88% hydrolyzed) was purchased from Sigma Chemical Co. (St Louis, MO, USA). Acetonitrile (ACN; HPLC grade) and Dichloromethane (DCM) were purchased from Rankem Fine Chemicals (New Delhi, India) and Merck Ltd. (Mumbai, India), respectively.

### Preparation of drug-loaded PLGA nanoparticles

The nanoformulations were made by emulsion diffusion evaporation method [25]. Nanoparticles of each drug were prepared separately. Briefly, the drug (20 mg) and the PLGA (20 mg) were dissolved separately in methanol (1 ml) and Dichloro methane (7 ml) respectively. The two solutions were mixed to get the oily phase, which was then added to Poly vinyl alcohol (PVA). PLGA nanoparticles containing the candidate drugs were optimized for different drug polymer ratios and concentration of PVA. The mixture was homogenized (IKA—T18 basic, Ultra-Turrax). The resulting emulsion was moderately stirred to evaporate the organic solvent. The solution obtained was centrifuged in cold centrifuge at 15,000 rpm, 4°C for 20 minutes (Sigma 3K30, Germany) with three subsequent washings with double distilled water. The pellet was resuspended and lyophilized (Christ, Alpha 1-2LD, Germany) for 48 hrs (Operating at pressure lower than 0.07 mbar and condenser temperatures lower than -55°C) until completely dry nanoparticle powder was obtained. These nanoparticles were characterized and drug loading was calculated as per the standard protocols.

### Characterization of drug-loaded nanoparticles

#### Candidate drugs estimation

The drugs were analyzed using HPLC (Shimadzu Pump LC-20AD, Prominence liquid chromatography) with SPD— 20A prominence UV-Vis detector. C-18 column (Hibar 250 x 4.6 Purospher STAR, RP-18e (5μm)) was used. The chromatographic conditions were standardized separately for each of the three drugs, and standard plots were obtained. The correlation coefficient (r2) for nanoformulation of each candidate drugs was 0.99. The HPLC method for the estimation of the three candidate drugs in combination was also standardized.[26] For the combination of amlodipine, hydrochlorothiazide and candesartan, all the three candidate drugs were mixed. The stock solution was used to make serial dilutions within the range of 100 to 2.5 μg/ml to get standard plot. The peaks were separated by adjusting the gradient in the mobile phase (potassium dihydrogen phosphate buffer PH = 6.0 & acetonitrile) at flow rate 1ml/min, injection volume of 20 μl and detection wave length (λmax) of 237 nm.

#### Size and polydispersity index (PDI)

Particle size analysis was carried out on Zetasizer Nano ZS (Malvern Instruments, Malvern, UK), based on dynamic light scattering technique (DLS). The nanoparticle powder was suspended in double distilled water and vortexed extensively before Size analysis. The Zetasizer gives the hydrodynamic particle size of the developed nanoparticles.[27]

#### Surface morphology

Surface characteristics and shape of the developed nanoparticles were studied with the help of transmission electron microscope (TEM).

#### Fourier Transform-Infrared (FT-IR) analysis

FT-IR analysis measures the selective absorption of wave emitted by the vibrational mode of specific band after exited state. The FT-IR spectrum of the developed PLGA nanoparticles was obtained using a Nicolet iS10, Thermoscientific FT-IR spectrometer. Pellets were made by mixing the nanoparticle powder with KBr, and these pellets were then scanned in the IR range from 400–4000 cm-1.

#### In-vitro drug release

The in-vitro release of the nanoparticles prepared was conducted by the dialysis membrane method.[28] Three dissolution mediums were used to study the in-vitro drug release pattern, simulated gastric fluid (SGF, pH 1.2), simulated intestinal fluid (SIF, pH 8.0) and phosphate buffer saline (PBS) for physiological pH of 7.4. Tween 80 was added to maintain ideal sink conditions. The study set was maintained at 37± 0.5°C and 100 rpm. 5 ml of the sample was withdrawn (and analyzed) and replenished with fresh media at different time points for 15 days. The study was done in triplicate for each of the three conditions and the average concentration was taken for evaluation.

#### Pharmacokinetics

Single dose pharmacokinetic (PK) studies nanoformulation were done in overnight fasted Wistar rats. Rat doses equivalent to human doses for the three drugs were calculated as per standard guidelines.[29]

Rat doses of the three candidate drugs for the ‘NanoFDC’ were:

Hydrochlorothiazide: 2mg/Kg BW
Amlodipine: 1mg/Kg BW
Candesartan: 1mg/Kg BW

We first conducted pharmacokinetic (PK) studies with individual drugs and nanoformulation of the individual drugs. This was followed by PK studies with the combined preparation (free) and the nanoFDC. For these studies, rats were bled at the following time-points: 0, 1, 2, 6, 12, and 24 hrs for the free drug group and 0, 1, 2, 6, 12, 24, 48, 72, 96, 120, 144 and 168 hrs for the nanoparticle group. Plasma was separated and kept at -80°C till estimation by HPLC as described above. Every effort was made to have at least 5–6 samples at each time point.

#### Individual drug pharmacokinetics

The animals were divided into two groups, one for free drug and the other for the nano-entrapped drug, each group was further divided into four candidate drug groups. Each group consisted of 6 animals.

#### NanoFDC pharmacokinetics

The individually formulated nanoparticles of all the three drugs were weighed, as per the calculated doses for rats, combined and suspended in double distilled water (DDW) to obtain a single formulation to be administered. Similarly, the free drugs were also combined and suspended in DDW. Next, the animals were divided into two groups with six animals in each group, one group to be administered the NanoFDC and the other, just the free drug combination. Drugs were administered by oral feeding tube (cannula) and samples were collected sublingually.

### Animals

Wistar rats of either sex weighing 200–250 g were obtained from Central Small Animal House Facility of our institute. They were provided with the standard pellet diet and water ad libitum. They were kept at 12/12h light/dark cycle under controlled temperature (20–22°C). No animals were sacrificed during this study. The study was approved by the Animal Ethics Committee (IAEC), of the Post Graduate Institute of Medical Education and Research (PGIMER), Chandigarh (UT), India.

### Statistical analysis

The drug entrapment, drug loading and the in vitro drug release are expressed as percentages. The nanoparticle sizes are expressed in nanometer scale. The FT-IR spectra shows the peaks at different wave numbers expressed as cm-1. Cmax and Tmax were obtained from actual plasma concentration versus time data. The area under the curve (AUC) was calculated using the trapezoidal rule, from the plasma vs. time plots. AUC0-t, AUC0-∞, total AUC and mean residence time (MRT) were calculated from the individual AUCs from the plasma-time plots. Results of PK studies are expressed as mean ± standard deviation (SD). The difference in PK parameters between the two groups was analyzed by student’s t-test and a p value of <0.05 was considered significant.

## Results

### Candidate drugs estimation, entrapment efficiency, drug loading and nanoparticle sizing

The drugs were characterized for their entrapment efficiency, drug loading and nanoparticle size. The optimized drug-polymer ratio was 1:1, with a PVA concentration of 1% for candesartan nanoformulation and 1.5% for the amlodipine and hydrochlorothiazide nanoformulations development. The entrapment efficiencies ranged from 29.34% for hydrochlorothiazide to 42% for amlodipine, and the drug loading from 18.05% for hydrochlorothiazide to 20% for amlodipine (Table 1). The average particle sizes as measured by zetasizer ranged from (570.9 nm- 898.5 nm) (Table 1). For each of the formulations TEM images were taken for particle size and surface morphology. These primary particle sizes were found to range from 110 nm to 180 nm. The PDI of the formulations were 0.810, 0.593 and 1.0 for candesartan, hydrochlorothiazide and amlodipine respectively.

**Table 1:** Characterization of the optimized nanoparticles (These values are means of a minimum of 5 batches).

Drug	Size of most frequently obtained particle (nm)	Average size(nm)	Entrapment(%)	Drug Loading(%)
Amlodipine besylate	171	643.3 ± 99	42.0	20
Candesartan	110–140	898.5 ± 108.2	33.5	21.09
Hydrochlorothiazide	180	570.9 ± 164.4	29.34	18.05

### Surface morphology

**Fig 1:**
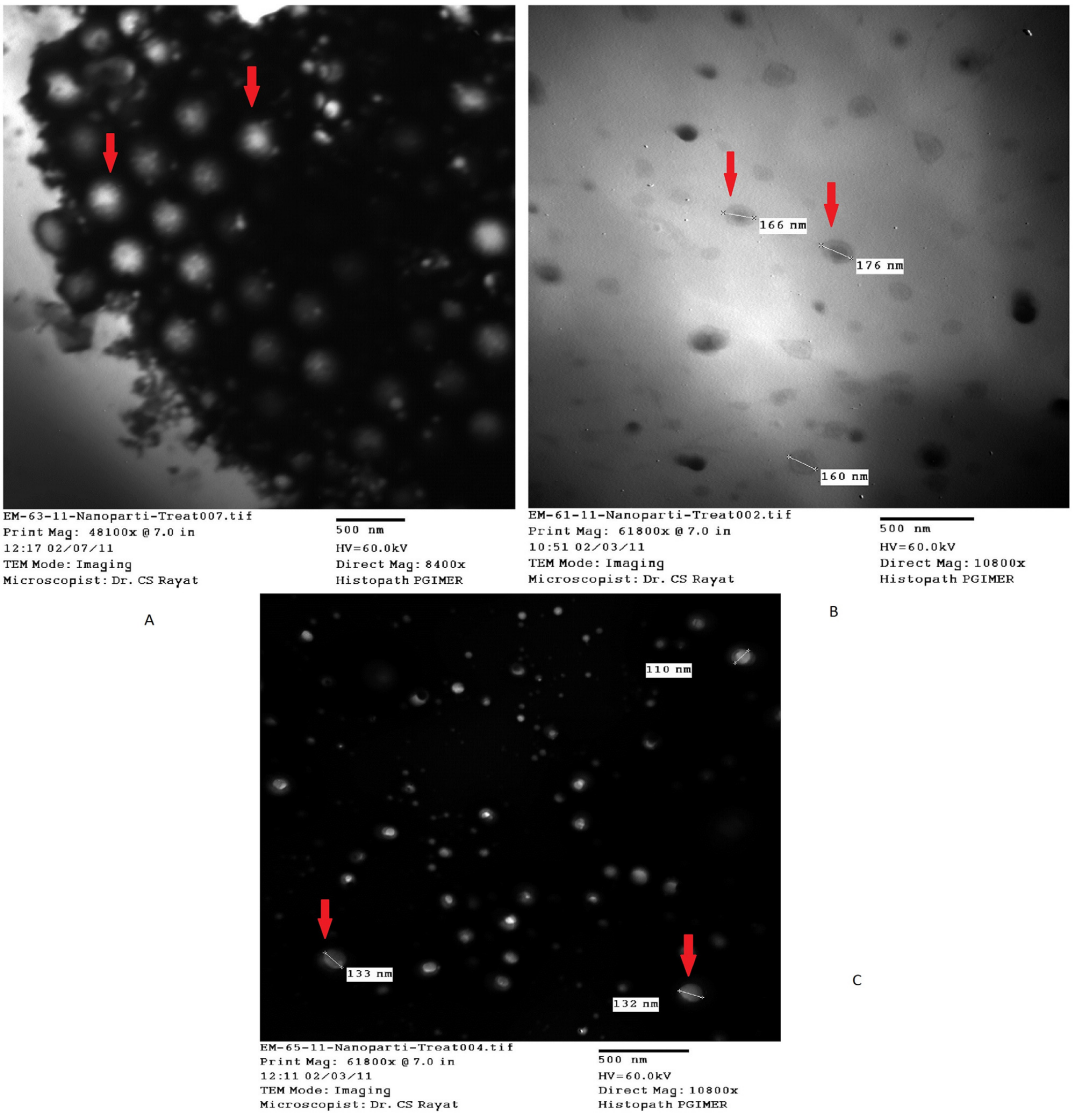
TEM images of (a) amlodipine (b) hydrochlorothiazide and (c) candesartan nanoparticles.

The developed nanoparticles showed smooth spherical surface morphology with the drugs entrapped inside the different nanoparticles when seen by the difference in the staining as observed under TEM (Fig 1).

### Fourier Transform-Infrared (FT-IR) analysis

**Fig 2:**
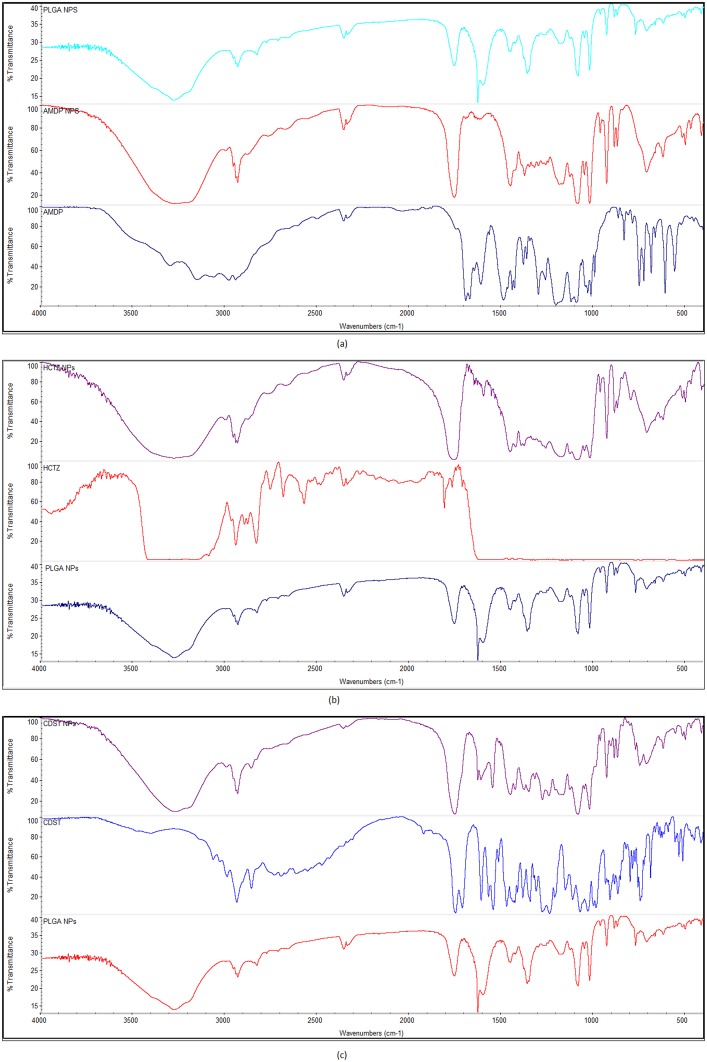
FT-IR spectra of (a) amlodipine (b) hydrochlorohiazide and (c) candesartan nanoparticles.

FTIR spectra of PLGA showed the peaks contributed by its function groups such as–C = O stretch of amide bond in the region of 1700–1800 cm-1, -OH stretching vibrations in the region of 3200–3600 cm-1, -CH stretch of CH2, -CH3 in the region of 2860–2940. FT-IR spectrum of Amlodipine showed the characteristic peaks contributed by function groups such as phenyl ring substitution in the region 1697–1699 cm-1and-H bonded OH in the region 3200–3300 cm-1. FTIR spectrum of Hydrochlorothiazide showed the characteristic spectrum contributes by the amine group in region 3500–3200 cm-11and-H bonded OH in the region 3200–3300 cm-1. FTIR spectrum of Candeartan showed the characteristic spectrum contributes by function group–CH aromatic and aliphatic stretch in region 3000–3100 cm-1 and 3000–2800 cm-1 respectively, -N-C- in region 1030–1281 cm-1, -C = O ester and acid stretch in region 1650–1755 cm-1 and 1650–1730 cm-1 respectively, and–C = C- (aromatic) stretch in region 1450–1500 cm-1. The FT-IR spectrum of the formulated PLGA nanoparticles showed no chemical interaction between the individual drugs and the polymer. The characteristic peaks of the PLGA polymer and the drugs could be observed in the drug entrapped nanoparticles showing that the drug is only physically entrapped inside the PLGA nanoparticles (Fig 2).

### In-vitro drug release

**Fig 3:**
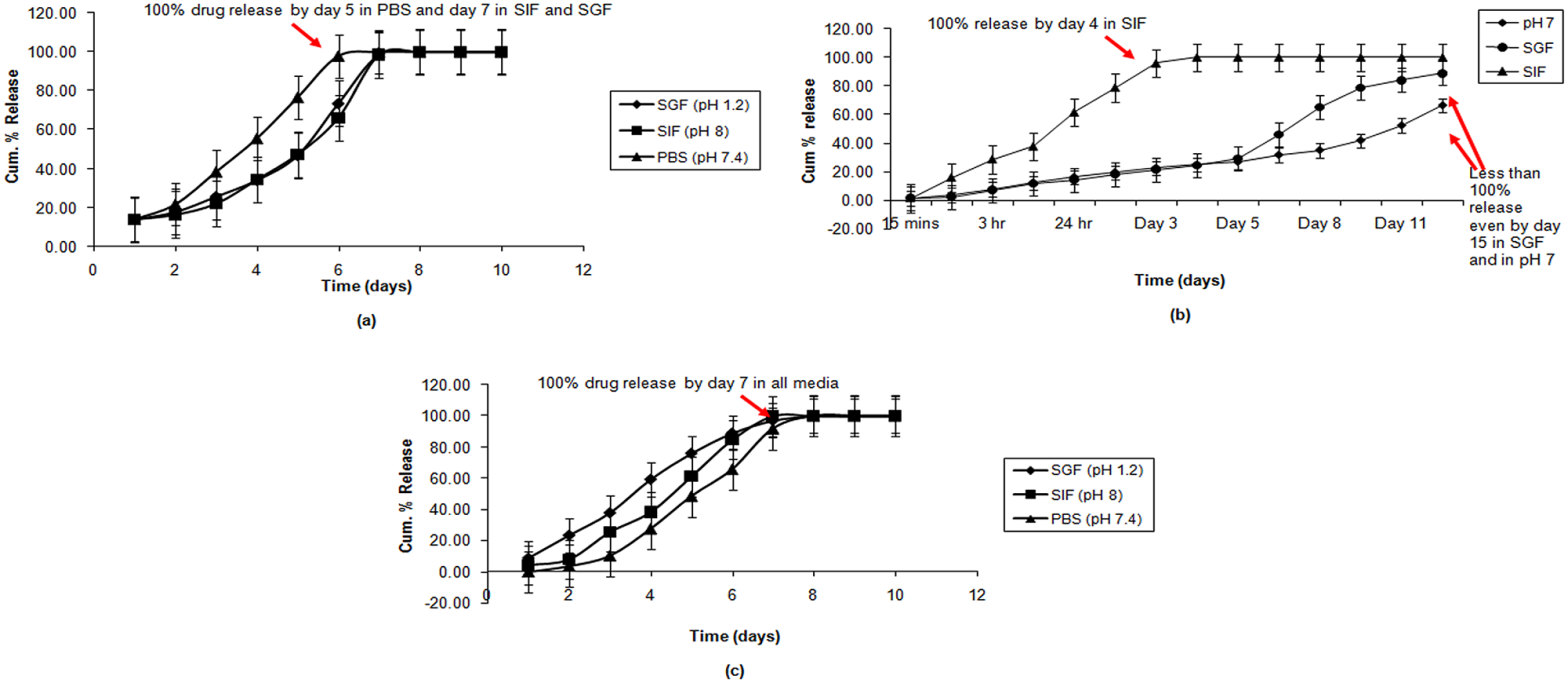
*In-vitro* drug release profiles of (a) amlodipine, (b) hydrochlorothiazide, and (c) candesartan.

The in vitro release study in the three different media showed a consistently sustained release pattern of the developed nanoparticle formulations. The release of the three components of the nanopill did not follow a uniformly similar pattern. 100% drug release ranged from 4 days (amlodipine and candesartan in SIF and SGF) to a maximum of 15 days (hydrochlorothiazide in PBS). (Fig 3)

### In-vivo release (Pharmacokinetics)

**Fig 4:**
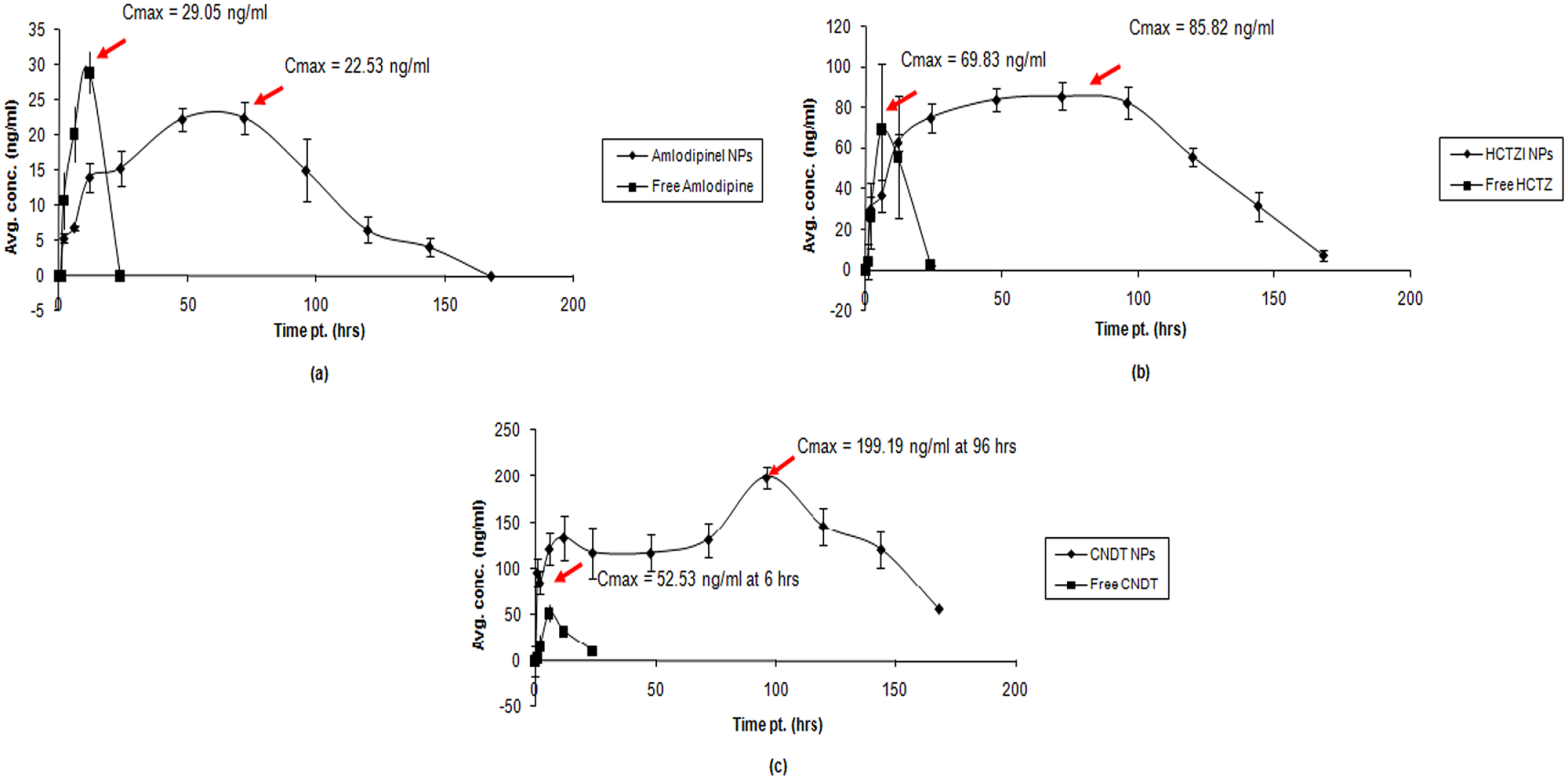
PK profiles of the developed nanoparticles vs. free drugs in rats (a) amlodipine, (b) hydrochlorothiazide, and (c) candesartan.

Plasma levels of all the three drugs were observed up to 144 hours (day 6) in the nanoparticle treated group, whereas only up to 24 hours in the free drugs group. (Fig 4).

**Fig 5:**
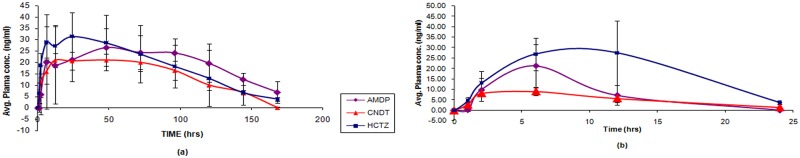
PK profile of: (a) The developed NanoFDC and (b) Combination of the free candidate drugs.

The pharmacokinetics of the NanoFDC (the combination of the nanoparticles of the three drugs) showed a sustained release of the three components for up to 6 days (144 hours). The PK parameters of the component drugs in the NanoFDC, show improved bioavailability (AUC 0-∞), compared to the free drugs when given in combination (Table 2 and Fig 5).

**Table 2:** Pharmacokinetic parameters of the individual components of the HTN NanoFDC in free and nanoparticles.

PK parameters	Amlodipine		Hydrochlorothiazide		Candesartan	
	Free	NP	Free	NP	Free	NP
**Cmax**	21.39±1.98	26.57±3.95 *	27.58±2.28	31.44±1.97 *	21.22±0.73	22.13±3.04 *
**Tmax(hrs)**	6±0.00	48±0.003 *	12±0.00	24±0.00 *	6±0.01	12±0.00 *
**AUC(0-∞)**	200.03±21.04	3573.08±87.3 *	488.81±9.38	3275.39±72.04 *	331.98±0.85	2621.41±32.45 *
**MRT(hrs)**	8.9±3.4	80.59±3.43 *	11±2.8	69.20±3.2 *	9±0.02	101.49±3.2 *
**AUMC**	1789.308±74.32	287966.87±12.75 *	5401.276±16.4	226666.59±3.93 *	3003.723±33.17	266052.72±213.03 *

Cmax—ng/ml; AUC—ng.hr/L;

* Free Vs Nanoparticles (NPs) significant at p < 0.05 (n = 5)

## Discussion

We have designed and developed a ‘nanoFDC’ for hypertension, containing nanoparticles of three antihypertensive drugs, amlodipine, hydrochlorothiazide and candesartan, combined in a fixed-dose combination. The candidate drugs chosen for the present nanoFDC were based on guidelines recommending their combined use, and various trials having proven their benefits.[30]

Our study has shown for the first time sustained levels lasting for nearly 7 days of the “NanoFDC” containing commonly co-prescribed drugs for hypertensive patients. A routine prescription for hypertension contains 2–3 drugs, which, in most cases, go on for the rest of the life. This chronic treatment regimen leads to poor adherence and therapeutic failure.[9] Our novel formulation has the potential to solve these problems to some extent.

The results of the pharmacokinetic study, similarly, show significant difference between the plasma drug levels over time between the free drugs and the nanoformulation. The increase in the AUC of the drugs administered as individual nanoparticles as well as in combination as in the NanoFDC, indicates an improvement in the drug bioavailability. This could be due to several reasons. The polymer, PLGA, has bioadhesive properties which bind the nanoparticles with the mucosa of the GI tract.[31] This intimacy of contact with the epithelial cells increases the MRT which could lead to a better drug absorption and reduced variability and erratic absorption into the blood.[31] The nanoparticles within the GIT are preferentially taken up by the M cells/enterocytes in the Payer’s patches of the GALT (gut associated lymphoid tissue), from where they are absorbed into the systemic circulation.[32] A possible mechanism enabling the particles to pass through gastrointestinal (and other physiological) barriers could be: paracellular passage—particles ‘‘kneading” between intestinal epithelial cells due to their extremely small size; endocytotic uptake—particles absorbed by intestinal enterocytes through endocytosis; and lymphatic uptake—particles adsorbed by M cells of the Peyer’s patches (particle size <5 μm).[33]

Bypassing the hepatic first-pass metabolism of the nanoparticles also contributes to the increased bioavailability of the drug. This phenomenon can be associated with the significantly increased AUCs of the NanoFDC studied and has been consistently seen by us in previous studies.[19, 20, 21, 24]

In vitro drug release of the three drugs was gradual and showed a 100% release by day 6. The polymer shows variable swelling at different times in different media, considering the different pH, which is why time to release 100% of the drug varies in all the three in vitro media. These results supported the in vivo experiments.

We have observed that all the three candidate drugs when entrapped inside PLGA nanoparticles behave in a similar manner individually as well as when these nanoparticles are combined into a single formulation. We have successfully shown a sustained release from a “nanopolypill” formulation combining nanoparticles of drugs for IHD also [24].

The physicochemical characterization of the designed nanoformulations included particle size analysis and chemical interaction studies. The sizes of the developed nanoparticles have been presented in two ways, as the primary size observed by TEM and the hydrodynamic size observed by the Zetasizer. Polydispersity index (PDI) tells us about the homogeneity of the nanoparticles and complements the information obtained from sizing studies.[15] the FT-IR confirmed no chemical interaction between the components of the nanoformulations.

Despite the advantages offered by FDCs and sustained release formulations, a number of limitations present with this kind of dosing. Problems like the lack of flexibility in dosage, side effects due to one of the components of the FDC, the interactions amongst the candidate drugs, and the need to withdraw one component due to its side effects etc. still need to be addressed. Keeping in mind that these problems exist, experiments for these novel formulations are must be designed in the early efficacy evaluation stages so as to address these issues. A comprehensive look into this issue is also required to be worked upon.

Another important limitation is that, where antihypertensive drugs need to be withdrawn immediately. For eg. a patient on vasodilator may develop septic shock. Maintaining blood pressure in this case can be problematic. This is true for all long acting, sustained release formulations, and is not specific to this nanoFDC. This is also not insurmountable as higher doses of vasopressors may need to be used in the septic shock example.

On the whole this novel nanoparticle drug delivery system seems to be a promising strategy to achieve the aim of a weekly dosing regimen. This has the potential to improve patient adherence thereby enhancing treatment efficacy. The efficacy of this strategy, however, needs to be tested further in pharmacodynamic studies, in animal models of hypertension, which are being initiated in our institute.

### Limitation of the study

This study does not include efficacy evaluation of the developed ‘NanoFDC’. They are needed to validate this data, and our next study on efficacy evaluation of the ‘NanoFDC’ has already begun. Due to certain in-house limitations, efficacy experiments could not be included in these set of experiments.

## Conclusion

We have shown for the first time that encapsulating amlodipine, hydrochlorothiazide and candesartan inside PLGA nanoparticles, and then combining them into a single formulation, the “NanoFDC”, is a feasible concept and such a FDC provides a sustained release in plasma for up to a week in rats. However, further work needs to be done to optimize the formulation and overcome some of the problems found in this preliminary study.
